# Containing outbreaks at their source: an illustrative example of partial economic returns to US global health security funding

**DOI:** 10.7189/jogh.16.03023

**Published:** 2026-08-17

**Authors:** Deliana Kostova, Jill Kuhn, Muhammad Jami Husain, Sarah Pallas, Walter Ochieng, Benjamin Park

**Affiliations:** Centers for Disease Control and Prevention, Atlanta, Georgia, USA

**Keywords:** global health security, pandemic preparedness, health financing, Ebola, outbreak containment, economic returns, US exports

## Abstract

Following the 2014–2016 West Africa Ebola epidemic, the US expanded funding for global health security (GHS) to strengthen outbreak prevention, detection, and response capacity across countries. In this viewpoint, we offer an illustrative comparison between one narrow category of economic benefit to the US – avoided losses in merchandise exports – and US GHS funding directed to the region between 2016 and 2023. Using a transparent accounting identity, we compared US export losses plausibly averted through the local containment of ten Ebola outbreaks during this period against estimated regional GHS appropriations of USD 2.52 billion, applying the 2014 losses as a benchmark and a range of scenarios to reflect uncertainty in the scale of disruption averted. Under the benchmark scenario, each USD of funding corresponded to approximately USD 4.64 in avoided export-related losses. This scoping exercise is intended to convey the order of magnitude of potential returns, rather than a measured economic return or causal attribution, and it deliberately excludes broader benefits. Even through this narrow trade-based lens, the findings suggest that the economic value of containing outbreaks abroad may be large enough to warrant consideration in U.S. budget discussions.

The 2014–2016 Ebola outbreak in West Africa began with a single death in Guinea. By March 2014, clusters were occurring near the country’s borders, and by August, the disease had spread across the region, with cases in Liberia, Sierra Leone, Nigeria, Mali, and Senegal. Eventually, it killed over 11,000 people, disrupted regional economies, and spread panic across borders. In the US, one traveler died after arriving from Liberia and two healthcare workers were infected, but survived. Although Ebola did not spread further within the US, the outbreak still imposed substantial domestic economic costs, with estimates pointing to a relative loss of USD 1.08 billion in merchandise exports due to a slowdown in trade with affected countries [1]. Other costs to the US during the outbreak included over USD 360 million spent on equipping US hospitals with Ebola treatment units [2] and over USD 5.4 billion in emergency Ebola funding appropriated by the US Congress [3].

In the aftermath of the West Africa Ebola outbreak, the US government launched a global health security (GHS) initiative in 2016 aimed at helping countries prevent, detect, and respond to future outbreaks before they spread across borders [4]. Since then, the US has invested billions in efforts to improve GHS, including in sub-Saharan Africa (SSA), where the threat of Ebola and other emerging pathogens remains high [5]. In practical terms, these funds support capacities such as event-based and indicator-based surveillance, laboratory diagnosis, epidemiologic workforce training, rapid response, emergency coordination, and reporting systems that can help identify and contain outbreaks earlier. While these efforts have contributed to strengthening the international health security infrastructure, questions persist about their return on investment and the benefits to the US.

Through this viewpoint, we sought to provide an illustrative comparison between one implied benefit of outbreak containment and congressionally appropriated GHS funding, using the setting of Ebola containment. The intended audience includes policymakers who make or inform decisions about global health and preparedness funding, for whom an implied economic return metric can complement epidemiologic and programmatic evidence by showing how investments in outbreak containment abroad have relevance for US economic outcomes.

Rather than estimating all benefits of GHS funding, we focus on one measurable category of potential benefit – avoided US merchandise export losses – to illustrate the plausible order of magnitude of returns. We used US merchandise export losses in this exercise for the following reasons: they are a directly US-relevant outcome; they have already been quantified for this specific type of event in a peer-reviewed study [1], providing a citable, transparent data point for a benchmark; and they constitute a deliberately narrow and well-defined measure, which suits a scoping exercise such as this. We likewise use Ebola as the setting for this exercise because it is the only epidemic occurrence for which a published US export-loss estimate exists and which has a documented history of cross-border spread. This single-disease, single-channel focus limits the risk of overstating the overall value of GHS funding. This estimate is intended as an explanatory policy benchmark, rather than a comprehensive cost-benefit analysis or a causal attribution of the effect of each GHS USD.

## APPROACH

We conducted a scoping exercise intended to demonstrate the order of magnitude of potential returns by comparing a narrow measure of benefits (US export losses plausibly averted by successful containment of Ebola outbreaks) to US government funding for GHS in SSA in 2016–2023. The expression below is an accounting identity that makes the underlying assumptions explicit, rather than a structural or predictive model.

Implied return metric = (number of contained Ebola outbreaks × implied averted US export losses per contained outbreak)/(total US GHS funding for SSA)

The benchmark event for avertable losses is the 2014 West Africa Ebola outbreak. In 2014, this outbreak resulted in the relative loss of USD1.08 billion (95% confidence interval (CI) = 1.078–1.081) in US exports as the virus spread across the borders of multiple countries in West Africa. The 95% CI, taken directly from our published source estimate [1] reflects only the point estimate’s statistical uncertainty and does not capture structural or model uncertainty representative of the potential impact of future outbreaks. For this reason, we do not rely on this confidence interval to characterise the structural uncertainty of the implied return metric, and instead create a range of alternative scenarios described below.

The 2014 estimate is appropriate as a historical benchmark because it represents known losses that can and have occurred when an outbreak is not rapidly contained within the borders of origin, and because it took place prior to the expansion of targeted GHS funding in 2016. Since the benchmark outbreak occurred in different countries than subsequent outbreaks, we treat its losses as representative of the SSA region, rather than country-specific. We acknowledge that the economic consequences of an epidemic depend on the affected countries’ specific trade relationships with the US, and that countries affected in 2014 are not interchangeable with the countries affected by later outbreaks. We therefore adopt a regional interpretation, ensuring that the denominator includes funding for the entire region to avoid overstating the implied return metric, and include a wide range of scenarios to absorb this cross-country heterogeneity rather than assume a single fixed loss per outbreak. To reflect uncertainty about the scale of export disruption potentially averted, we estimated the implied return metric under alternative scenarios in which each contained outbreak is associated with 10%, 25%, 50%, or 100% of the historical benchmark export losses.

Between 2016 and 2023, ten Ebola outbreaks occurred in Africa (Uganda, Guinea, and the Democratic Republic of the Congo) [6]. All were locally contained within country borders [7]. Under the benchmark 100% scenario, the implied averted US export losses equal USD 11.68 billion (95% CI = 11.67–11.70) in constant 2024 USD. Under the 10%, 25% and 50% sensitivity scenarios, the implied averted losses are USD 1.168 billion (95% CI = 1.167–1.170), USD 2.921 (95% CI = 2.918–2.924), and USD 5.842 (95% CI = 5.835–5.848), respectively.

The ‘investment’ denominator is based on publicly available data on US government funding for GHS programmes. Between 2016 and 2023, the US government appropriated USD 5.645 billion for GHS [5]. The fraction of these funds that was allocated to SSA is not readily available; we estimate it as the SSA share of total US bilateral aid for health sector programme support, which has an average of 42% (range = 33–50%) for the period [8]. We use this health-aid distribution as a proxy because a published SSA-specific breakdown of GHS appropriations is not available, and because US GHS programming is heavily concentrated in African priority countries, making the regional health-aid distribution a reasonable approximation. We treat the SSA share as a key uncertain parameter rather than a fixed value: the 33–50% range is carried through the calculation, and combined with the published export-loss interval it generates the reported bounds on the comparison. This results in an estimated GHS funding for SSA of USD 2.52 billion (range 1.99–3.04). We use SSA-directed GHS funding because both the benchmark losses and the containment scenarios are interpreted at the regional rather than country level. All values were adjusted to constant 2024 USD using the US gross domestic product deflator and are provided in the **Online Supplementary Document**.

## FINDINGS

The benchmark scenario compares USD 11.68 billion (95% CI = 11.67–11.70) in potentially averted US export losses with USD 2.52 billion (1.99–3.04) in estimated US GHS investment in SSA. This comparison implies that each dollar of funding during this period corresponded to USD 4.64 (3.84–5.85) in avoided export-related losses. Across alternative assumptions about the scale of averted export losses, the estimated implied return metric ranges from USD 0.46 in the lowest-impact scenario to USD 2.32 in the scenario of 50% impact (**Table 1**).

**Table 1:** Comparing US GHS funding, SSA, 2016–2023, to implied averted exports losses under alternative assumptions

	Billions, constant USD in 2024		
**Scenario**	**Implied averted losses, US merchandise exports**	**Estimated US GHS funding**	**Implied return metric**
10% of benchmark	1.17	2.52	0.46
25% of benchmark	2.92	2.52	1.16
50% of benchmark	5.84	2.52	2.32
100% of benchmark	11.68	2.52	4.64

GHS – global health security, SSA – sub-Saharan Africa

## DISCUSSION

By focusing on Ebola, a disease with documented economic consequences for the US and a history of cross-border spread, we demonstrate how investments in GHS can correspond to measurable economic benefits to the US when outbreaks are contained before expanding across borders. Funding for GHS supports capacities for prevention, detection, and response, including surveillance, laboratory diagnosis, workforce preparedness, and emergency coordination. In the benchmark scenario, our estimate suggests that a USD 1 investment compares to more than USD 4 in avoided export-related losses. This implied return takes a narrow lens and does not contain other benefits to the US, such as avoided domestic hospital costs or averted outbreaks other than Ebola, such as Marburg and Lassa. For policymakers and public health leaders, the value of this estimate lies less in the precise numerical ratio than in the demonstration that the economic benefits of outbreak containment abroad may be large enough to matter in budget discussions. Even when viewed through a narrow trade-based lens, GHS investments can be understood not only as health protection, but also as a strategy to reduce downstream economic disruption to the US.

Many limitations are inherent to illustrative estimates that simplify complex pathways in order to provide a transparent benchmark. First, this analysis is based on a simple comparison and is not intended to causally attribute containment outcomes to US GHS funding; outbreak trajectories also reflect country context, pathogen characteristics, domestic response, and support from multiple partners. Several factors other than US GHS funding could have contributed to improved containment after 2014, including lessons learned from prior epidemics, investments by national governments and other funders, advances in diagnostics, and the development and deployment of Ebola vaccines. Our implied returns metric does not aim to isolate the US GHS contribution from others but simply to compare a narrow measure of gains to contributions. Second, there is inherent uncertainty about the applicability of a benchmark event to subsequent outbreaks; we therefore present it as one scenario rather than the only plausible estimate. Third, the denominator relies on a proxy for the SSA share of GHS funding drawn from the distribution of general bilateral health aid, which may not match the true geographic allocation of GHS resources and thus introduces additional uncertainty into the comparison. Fourth, merchandise exports are only one narrow channel of economic impact; readers should not infer that export losses are the principal economic consequence of epidemics. The narrow lens of the calculation intentionally excludes broader and less easily quantified benefits, such as the avoided costs of US hospital preparedness, reductions in mortality and morbidity, economic benefits to African countries, or improvements in regional stability, thus understating the full value of outbreak prevention.

## CONCLUSIONS

Ten Ebola outbreaks were locally contained in Africa between 2016 and 2023, a period of expanded US funding for GHS to the region. We compared this funding with one specific potential benefit to the US: the avertable US export losses from outbreak containment, using losses from the 2014 West Africa Ebola epidemic as a benchmark. Under the assumptions specified here, the comparison resulted in an implied return metric on the order of four dollars in implied benefit per dollar of funding. This exercise illustrates the plausible order of magnitude of one category of return and suggests that the economic benefits of outbreak containment abroad may be large enough to warrant consideration in budget discussions.

## Additional material


Online Supplementary Document

## Data Availability

**Data availability:** All data in this scoping exercise are available in the **Online Supplementary Document**.
